# Molecular Therapies in Cardiovascular Diseases: Small Interfering RNA in Atherosclerosis, Heart Failure, and Hypertension

**DOI:** 10.3390/ijms25010328

**Published:** 2023-12-26

**Authors:** Riccardo Sarzani, Francesco Spannella, Chiara Di Pentima, Federico Giulietti, Matteo Landolfo, Massimiliano Allevi

**Affiliations:** 1 Internal Medicine and Geriatrics, ESH “Hypertension Excellence Centre”, SISA LIPIGEN Centre, IRCCS INRCA, 60127 Ancona, Italy; r.sarzani@univpm.it (R.S.); m.landolfo@inrca.it (M.L.);; 2 Department of Clinical and Molecular Sciences, University “Politecnica delle Marche”, 60126 Ancona, Italy

**Keywords:** small interfering RNA, atherosclerosis, heart failure, hypertension, amyloidosis, inclisiran, olpasiran, patisiran, zilebesiran, lepodisiran

## Abstract

Small interfering RNA (siRNA) represents a novel, fascinating therapeutic strategy that allows for selective reduction in the production of a specific protein through RNA interference. In the cardiovascular (CV) field, several siRNAs have been developed in the last decade. Inclisiran has been shown to significantly reduce low-density lipoprotein cholesterol (LDL-C) circulating levels with a reassuring safety profile, also in older patients, by hampering proprotein convertase subtilisin/kexin type 9 (PCSK9) production. Olpasiran, directed against apolipoprotein(a) mRNA, prevents the assembly of lipoprotein(a) [Lp(a)] particles, a lipoprotein linked to an increased risk of ischemic CV disease and heart valve damage. Patisiran, binding transthyretin (TTR) mRNA, has demonstrated an ability to improve heart failure and polyneuropathy in patients with TTR amyloidosis, even in older patients with wild-type form. Zilebesiran, designed to reduce angiotensinogen secretion, significantly decreases systolic and diastolic blood pressure (BP). Thanks to their effectiveness, safety, and tolerability profile, and with a very low number of administrations in a year, thus overcoming adherence issues, these novel drugs are the leaders of a new era in molecular therapies for CV diseases.

## 1. Introduction

**Figure 1:**
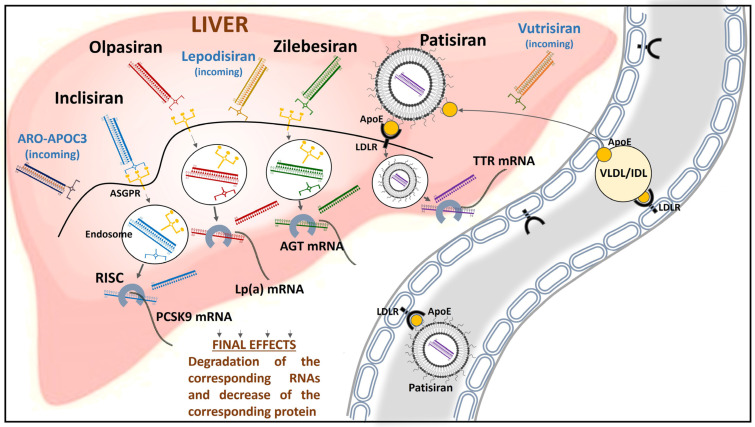
Mechanism of hepatic delivery and RNA interference of siRNAs. All the siRNAs discussed in the text exert their action within the liver, but they use different delivery mechanisms. Inclisiran, olpasiran, lepodisiran, ARO-APOC3, and zilebesiran are administered subcutaneously, covalently linked to a GalNAc “trident” that binds with high affinity to the ASGPR on the hepatocyte surface. Hence, they gain endosomal entry into the cell and dissociate from GalNAc and ASGPR inside the endosome. Patisiran is administered intravenously, and it is formulated as an encapsulated lipid nanoparticle consisting of a largely hydrophobic core with inverted micelles of lipids and an outer coating of PEG lipids and cholesterol-acquiring ApoE from circulating VLDL and IDL. Patisiran nanoparticle coated by ApoE binds to the LDLR on the hepatocyte surface, and internalization by endocytosis ensues. Once inside the cell, siRNAs bind a specific protein complex and form the RISC, where they are separated into two strands: one of these strands (the “guide” strand) binds its target, a specific mRNA sequence, and leads to its degradation, preventing RNA translation and inhibiting corresponding protein synthesis. The specific targets are the following: PCSK9 for inclisiran, Lp(a) for olpasiran (and lepodisiran), APOC3 for ARO-APOC3, TTR for patisiran (and vutrisiran), and AGT for zilebesiran. AGT: angiotensinogen; APOC3: apolipoprotein C-III; ApoE: apolipoprotein E; ASGPR: asialoglycoprotein receptor; GalNAc: N-acetyl-galactosamine; IDL: intermediate-density lipoprotein; LDLR: low-density lipoprotein receptor; Lp(a): lipoprotein(a); PCSK9: proprotein convertase subtilisin/kexin type 9; PEG: polyethylene glycol; RISC: RNA-induced silencing complex; siRNA: small interfering RNA; TTR: transthyretin; VLDL: very low-density lipoprotein.

Small interfering RNA (siRNA) represents a novel therapeutic strategy leading to an increasing number of new drugs, or better, a completely novel armamentarium that selectively reduces the production of a specific protein. RNA interference (RNAi) is a regular cellular process of gene silencing, a post-transcriptional cellular control of gene expression that today represents one of the most promising and rapidly advancing frontiers in biology and drug development [1]. Its discovery has been claimed as “a major scientific breakthrough that happens once every decade or so”, and was recognized with the award of the 2006 Nobel Prize for Physiology or Medicine [2]. By harnessing the natural biological process of RNAi occurring in our cells, a new class of drugs known as siRNA is now a reality. In the cardiovascular (CV) field, several siRNAs have been developed in the last decade and tested in clinical trials (Table 1). These modified RNA molecules are designed for specific messenger RNA (mRNA) that resist digestion by ribonucleases and (except for patisiran, as discussed below) are covalently linked to an N-acetyl-galactosamine (GalNAc) “trident” that binds with high affinity to the hepatic asialoglycoprotein receptor (ASGPR) and therefore is quickly taken up by the liver from the circulation, resulting in it being undetectable about 48 h after administration [3]. Once in the liver, these double-strand RNA molecules bind to a specific protein complex, which is present as a post-translational regulatory mechanism in any cell, and form the RNA-induced silencing complex (RISC) where they are separated into two strands: one of these strands (the “guide” strand) binds to a specific (“target”) mRNA sequence. Cleavage and degradation of the target mRNA ensue, preventing translation and specific protein synthesis. The other strand (the passenger RNA) is degraded during or after the formation of the RISC (Figure 1). Although these drugs exert their effects on the liver, they proved to be safe even in various degrees of hepatic impairment, as discussed below. The aim of this focused narrative review is to present in detail how the first four siRNAs (inclisiran, olpasiran, patisiran, and zilebesiran), specifically designed to prevent CV diseases, are going to be an effective and fascinating instrument in the great struggle against the first cause of death worldwide. 

## 2. Inclisiran

Inclisiran has been the first siRNA to obtain the approval of the Food and Drug Administration (FDA) and the European Medicines Agency (EMA), and it is prescribed already in clinical practice to reduce the hepatic proprotein convertase subtilisin/kexin type 9 (PCSK9) production [4]. PCSK9 is a protease, mainly synthesized in the hepatocytes, that regulates the metabolism of the low-density lipoprotein receptor (LDLR) [5], leading to increased circulating levels of low-density lipoprotein cholesterol (LDL-C) strongly associated with the incidence of atherosclerotic CV disease (ASCVD) [6]. The two strands of modified RNA are linked to the GalNAc “trident” so that inclisiran can be rapidly captured by hepatic ASGPR after subcutaneous injection. Once it has gained endosomal entry into hepatocytes, inclisiran binds to RISC and leads to PCSK9 mRNA degradation, thus hampering PCSK9 synthesis and secretion [7] (Figure 1). PCSK9 circulating levels showed a mean 70% reduction after 60 min of drug infusion [8]. Inclisiran is no longer detected in the bloodstream 48 h after administration; therefore, dose adjustments are not needed in patients with mild-to-severe chronic kidney disease [9] and patients with mild-to-moderate liver disease [10]. The ORION-10 trial conducted in patients with ASCVD found a change in LDL-C of 52.3% at day 510 in those taking inclisiran (284 mg on day 1, then after three months and every 6 months thereafter over a total duration of 540 days) compared to a change of 1% in those taking placebo (*p* < 0.001). Even in patients having equivalent ASCVD risk, with no history of ASCVD, the ORION-11 trial found a greater change in LDL-C in those treated with inclisiran in comparison with placebo (49.9% vs. 4%; *p* < 0.001) [11]. The ORION-9 trial evaluated both the efficacy and the safety of inclisiran in heterozygous familial hypercholesterolemia (HeFH), finding a significantly lower LDL-C in the inclisiran group (300 mg) compared to placebo (−39.7% vs. −8.2%, *p* < 0.001) from baseline to day 510, irrespectively of HeFH genotype. Inclisiran also reduced lipoprotein(a) [Lp(a)] (−17.2% from baseline), another risk factor independently related to ASCVD [12]. Meta-analytic data from these three trials (ORION-9, -10, and -11) found a 24% lower rate of major adverse CV events (MACE) in patients treated with inclisiran (RR = 0.76; 95% CI, 0.61–0.94, *p* = 0.01) [13]. However, other meta-analytic data from ORION-1, -9, -10, and -11 did not confirm these findings, given that fatal and nonfatal myocardial infarction (RR 0.85, 95% CI, 0.37–1.95), fatal and nonfatal stroke (RR 0.69, 95% CI, 0.11–4.21), and CV mortality (RR 1.11, 95% CI, 0.56–2.21) were not significantly reduced with inclisiran compared to placebo [14]. Whether the LDL-C lowering found with inclisiran will lead to reduced CV events will be clarified by ongoing outcome studies. Indeed, the impact of inclisiran on CV outcomes in the long term will be evaluated in both the HPS-4/TIMI 65/ORION-4 trial (estimated completion in July 2026) and VICTORION-2P trial (estimated completion in October 2027). As suggested by Mendelian randomization studies, the effect of LDL-C on ASCVD risk increases with increasing exposure duration; a 5-year lipid-lowering treatment leads to a nearly 25% reduction in the relative risk of ASCVD for every mmol/L (corresponding to 38.7 mg/dL) of LDL-C decrease, while a 55% reduction in ASCVD risk is expected after a 52-year exposure to lower LDL-C per mmol/L of LDL-C decrease [15]. 

The safety of inclisiran has also been evaluated in ORION-9, -10, and -11 trials, with findings showing an injection site reaction (ISR) to be the most common adverse event (5% incidence in the inclisiran arm). Only 2% and 2.5% of patients taking inclisiran in the ORION-10 and ORION-11 trials, respectively, developed antibodies against the drug [11], with no differences in clinical efficacy and safety, regardless of their age [16]. We have recently published a peculiar case of a female patient affected by HeFH and very high levels of LDL-C who developed a relapsing painful, delayed, cell-mediated hypersensitivity reaction at the injection site to both alirocumab and evolocumab, two human monoclonal antibodies (mAbs) administered in daily clinical practice to inhibit PCSK9 [17]. This reaction was likely caused by polysorbates (PS20 and PS80), which are typical excipients used to stabilize biological formulations. Protein aggregates can form after the degradation of PS20 or PS80, thus potentially stimulating immunogenicity through the direct activation of complement, leading to a type IV immunological reaction [18,19]. In this patient, treatment was switched to inclisiran, which was well tolerated, being not a protein and not needing that kind of excipient. As a follow-up of this case, the patient became pregnant 11 weeks after the second administration of inclisiran, thus representing to date the first case of pregnancy initiated after inclisiran injection. The pregnancy is monitored and goes on without problems, even if the patient’s LDL-C levels have been substantially increased after lipid-lowering treatment withdrawal, as expected. 

## 3. Olpasiran

Lp(a) is a lipoprotein associated with an increased risk of ischemic heart disease [20], causally involved in atherosclerosis [21,22] and calcific valvular aortic stenosis [23]. The apolipoprotein(a) gene (LPA) regulates its expression [24], and its circulating levels are mostly genetically determined (up to 90%) [20]. Although Lp(a) is a known cause of atherosclerosis and cardiac valvular damage, until now, only mAbs-targeting PCSK9 and inclisiran have been found to reduce its levels quite modestly [25]. In this context, pelacarsen, an antisense oligonucleotide against LPA mRNA, is still under investigation in a phase 3 trial [Lp(a)HORIZON, NCT04023552] and might be used in clinical practice before olpasiran [26]. Olpasiran is the first siRNA designed to reduce apolipoprotein(a) mRNA in the liver, with published clinical studies in phases 1 and 2. After subcutaneous injection, it is directed to the liver and conjugated to GalNAc, as well as inclisiran. Inside the hepatocytes, it is assembled into RISC and binds apolipoprotein(a) mRNA, causing its degradation so that Lp(a) cannot be produced (Figure 1). A single dose of olpasiran led to dose-dependent lower and sustained Lp(a) levels up to 6 months in a phase 1 study [27]. In the phase 2 OCEAN(a)-DOSE trial [28], 281 patients with a screening serum Lp(a) > 150 nmol/L (~70 mg/dL) and a history of ASCVD were randomized to subcutaneous olpasiran (either 10 mg, 75 mg, or 225 mg every 12 weeks, or 225 mg every 24 weeks) versus placebo for 48 weeks, and then followed for a minimum of 24 weeks in a safety follow-up. At 36 weeks, serum Lp(a) levels were significantly reduced in the olpasiran group in a dose-dependent manner, reaching a mean percent change of −101.1% with the 225 mg dose administered every 12 weeks (all *p* < 0.001) without a significant increase in the incidence of adverse events across the groups; painful ISR was the most prevalent adverse event observed with olpasiran. Data on its safety in patients with mild and moderate hepatic impairment will be available soon, thanks to a specific trial completed (NCT05481411). As announced during the latest European Society of Cardiology (ESC) congress (O’Donoghue ML et al., ESC Congress 2023), patients taking ≥75 mg of olpasiran in the previous year maintained nearly a 50% Lp(a) reduction after the last administered dose. Furthermore, this study found olpasiran to affect oxidized phospholipids on apolipoprotein B-100 (OxPL-apoB), a biomarker strongly associated with ASCVD [29]. Indeed, olpasiran may confer a dose-dependent reduction in OxPL-apoB levels, with a mean percent change of −104.7% in patients taking 225 mg of the drug every 12 weeks. The OCEAN(a)-Outcomes trial (NCT05581303) will investigate the long-term clinical efficacy and safety of olpasiran. Very recently, data of another siRNA, designed to reduce apolipoprotein(a) mRNA (Lepodisiran), have been published in a phase 1 study on 48 participants with elevated Lp(a) levels, finding a dose-dependent reduction in serum Lp(a) concentrations up to −97% with the highest dose (608 mg) [30]. 

In November 2023, another siRNA made its entrance to the scene of lipid-lowering molecular therapies: ARO-APOC3, a hepatocyte-targeting siRNA designed for the treatment of hypertriglyceridemia by hampering apolipoprotein C-III (APOC3) mRNA expression, a lipoprotein that inhibits triglyceride clearance by reducing lipoprotein lipase-mediated hydrolysis and hepatocyte uptake of triglyceride-rich lipoproteins [31]. In a phase I study on 112 patients [52 healthy volunteers with triglycerides (TG) > 80 mg/dL, 40 patients affected by hypertriglyceridemia (TG > 300 mg/dL), and 20 patients affected by chylomicronemia (TG > 880 mg/dL)], ARO-APOC3 was administered to 88 participants, while the placebo was administered to the remaining 24 participants by the subcutaneous route, either once or twice, with doses given on day 1 and day 29. The objective of this trial was to assess the safety, pharmacokinetics, and pharmacodynamic effects of ARO-APOC3 treatment. The most common adverse event was ISR; no adverse effects led to the discontinuation of the study drug [32]. Moreover, no adverse events related to thrombocytopenia were found, unlike what has been reported for volanesorsen, an antisense oligonucleotide-targeting APOC3 mRNA that showed results associated with thrombocytopenia in 76% of treated patients [33]. In the hypertriglyceridemia cohorts, both APOC3 and triglyceride levels were significantly reduced.

## 4. Patisiran (and Vutrisiran)

Transthyretin amyloidosis is a rare disease caused by the abnormal accumulation of transthyretin (ATTR) with subsequent morphological and functional changes in infiltrated tissues [34]. Almost all serum transthyretin (TTR) is synthesized and secreted by the liver and circulates as a tetramer, acting as a vehicle for the transportation of retinol and thyroxine. ATTR occurs secondary to the misfolding of TTR tetramer [35] and is classified as wild-type (wtATTR) (associated with age-related modifications) or mutated/variant (vATTR) (secondary to an inherited mutation in the TTR gene) [36]. Although traditionally considered a rare disease, ATTR-cardiomyopathy is increasingly being diagnosed and is emerging as an important under-recognized cause of heart failure (HF) in older adults, especially with preserved ejection fraction (HFpEF) [37]. Cardiomyopathy is one of the most relevant clinical features in ATTR amyloidosis; it is an infiltrative and restrictive cardiomyopathy characterized by increased biventricular wall thickness and reduced myocardial compliance that, in the first phase, results in increased left ventricular filling pressures and diastolic dysfunction, while later ends in systolic impairment [38]. A frequent extracardiac manifestation of the disease is polyneuropathy, presenting as a progressive and debilitating sensory-motor peripheral polyneuropathy and/or autonomic neuropathy [38]. Until recent times, liver transplantation or combined heart–liver transplantation were the only disease-modifying disposable treatments in ATTR [39]. Nevertheless, in the last decade, several new pharmacological treatments have been developed to treat ATTR amyloidosis [40]. Patisiran was the first commercialized siRNA, having received approval from the FDA in August 2018 [41]. Unlike inclisiran, olpasiran, and zilebesiran, which are GalNAc-conjugated and designed to be taken up by hepatic ASGPR, patisiran is taken up mainly by the liver through a different mechanism. It is administered as an intravenous infusion of 0.3 mg/kg every 3 weeks [42], it has an elimination half-life of 3 ± 2 days, and its pharmacokinetics are not affected by mild or moderate renal impairment and mild hepatic impairment [43]. It is formulated as an encapsulated lipid nanoparticle that protects therapeutic oligonucleotides from degradation by endogenous enzymes. The structure of this lipid nanoparticle consists of a largely hydrophobic core with inverted micelles of lipids that encapsulate the siRNA molecule, which is surrounded by an outer coating of polyethylene glycol (PEG) lipids and cholesterol that provide physicochemical stability in the circulation after intravenous administration [44]. The nanoparticle is then opsonized by apolipoprotein E (ApoE) taken from circulating lipoproteins, mostly very low-density lipoproteins (VLDL) and intermediate-density lipoproteins (IDL). The particle enters the liver through vascular fenestrations of endothelium and binds LDLR on the hepatocytes surface through ApoE, being thereby internalized by endocytosis [45]. Inside the cells, lipid nanoparticle structure is perturbed, resulting in siRNA molecule release that is complexed to the RISC and binds to the TTR mRNA, with a mechanism of action similar to that of the previously described siRNAs (Figure 1). In phase I and II studies, patisiran showed to reduce safely and effectively serum TTR [46]. The APOLLO phase III trial is a randomized, double-blind, placebo-controlled study involving patients with ATTR-polyneuropathy and is designed to evaluate the efficacy and safety of patisiran [47]. Furthermore, a subgroup of 126 patients with associated cardiac involvement, defined by the presence of a left ventricular wall thickness ≥13 mm without known arterial hypertension or significant aortic valve stenosis, was evaluated in a post-hoc analysis [48]. Patisiran was demonstrated to reduce mean left ventricular wall thickness, improve global longitudinal strain and cardiac output, lead to increased end-diastolic volume at month 18, and reduce N-terminal pro-B-type natriuretic peptide (NT-proBNP) levels at 9 and 18 months. Furthermore, patients in the treatment group had a 46% reduction in the rate of hospitalizations due to CV causes and all-cause death compared with those receiving placebo. Similar results on the possible efficacy of patisiran in improving cardiac parameters were also confirmed in real-life evidence [49]. The whole burden of amyloidosis also includes extracardiac symptoms. In the APOLLO trial, patisiran also showed good efficacy in improving symptoms related to extracardiac involvement, such as gastrointestinal. Patients taking patisiran had a 3.5-fold higher likelihood of diarrhea improvement versus patients taking a placebo (18% vs. 5%, respectively) after 18 months. A recently published interim 12-month analysis of the global OLE study, including patients from the APOLLO study and phase 2 OLE study, demonstrated that patisiran maintains long-term efficacy in patients with vATTR amyloidosis and polyneuropathy [50]. A total of 62 patients aged 65 years and older, including nine patients aged 75 years and older, received patisiran treatment in this placebo-controlled study. Dose adjustment was unnecessary for older patients, and there were no notable differences in safety or effectiveness between different age groups [51]. In the phase III APOLLO-B trial, 360 patients affected by cardiomyopathy were randomized to evaluate the efficacy and safety of patisiran [52]. This trial enrolled patients with both wtATTR and vATTR amyloidosis who had a history of HF and elevated NT-proBNP levels, ranging from 300 ng/L to 8.500 ng/L. Patisiran treatment resulted in a statistically significant improvement in the 6 min walking test (6MWT) compared to the placebo at 12 months. Patisiran also met the first secondary endpoint of improvement in quality of life as assessed with the Kansas City Cardiomyopathy Questionnaire (KCCQ). However, there was no difference between the groups in the time to the first event (all-cause hospitalization, urgent HF visits, or death), presumably due to the short duration of the study. In terms of clinical safety, the main risks associated with patisiran are reduced vitamin A levels (due to reduced transthyretin) and infusion-related reactions triggered by nanostructured siRNAs, which are minimized by premedication with intravenous corticosteroids (dexamethasone) and antagonists of histamine H1 and H2 receptors (diphenhydramine and ranitidine, respectively), in association with oral acetaminophen [53]. In the last few years, several data from real-life experiences with patisiran have confirmed its efficacy. Indeed, the median survival of 105 patients with ATTR-polyneuropathy taking disease-modifying drugs (including patisiran) was found to be significantly longer compared to untreated patients (12 years vs. 8 years) in a retrospective study conducted in Italy [54]. In another study, 15 patients affected by vATTR amyloidosis (mean age: 66.4 ± 7.8 years) were evaluated before starting therapy with patisiran and after 9 months of follow-up. Body composition, evaluated with bioimpedance analysis, changed significantly after 9 months of treatment, with an increase in fat-free mass, body cell mass, and body weight and a decrease in fat mass. A significant increase was observed also for the 6 MWT [55]. Other case reports of patients affected by vATTR amyloidosis treated with patisiran have corroborated the evidence of its efficacy in improving both neurological and CV symptoms in the real world [49]. 

Another siRNA has recently been approved for the treatment of ATTR-polyneuropathy. Vutrisiran, a second-generation GalNAc-conjugated siRNA binding ASGPR expressed on the surface of hepatocytes, has already demonstrated its safety and efficacy in ATTR-polyneuropathy (HELIOS-A trial) with a 3-monthly subcutaneous injection [56]. More specifically, it has determined a statistically significant improvement in polyneuropathy [evaluated with modified Neuropathy Impairment Score +7 (mNIS + 7)], quality of life, gait speed, nutritional status, and disability, compared with an external placebo. The ongoing double-blind, placebo-controlled, randomized HELIOS-B trial is evaluating its efficacy for the treatment of ATTR-cardiomyopathy, aiming at reducing the composite of all-cause mortality and recurrent CV events (CV hospitalizations and urgent HF visits) at 30–36 months. Full results are expected in early 2024 (NCT04153149).

## 5. Zilebesiran

Hypertension affects nearly 1.3 billion adults worldwide, and it is one of the most common risk factors for death [57]. Despite the availability of several new drugs and fixed-dose combinations [58,59], blood pressure (BP) control is achieved in less than a quarter of hypertensives worldwide [60]. One of the main problems is the adherence to drug therapy due to multi-pill regimens, too often not simplified by the use of single-pill combinations, that should be taken each day for decades. Several drugs are not effective for 24 h because of their pharmacokinetic and pharmacodynamic characteristics [61] or because they are taken at lower doses than recommended. Thus, higher BP can persist, especially during night-time and around awakening, with an important long-term variability strongly associated with CV events [62]. Therefore, drugs able to control BP for 24 h and day-by-day for months after a single administration could be the ideal therapies for most hypertensive patients. In this context, the interest has been focused on angiotensinogen (AGT), which is the only precursor of all angiotensin peptides and is mostly produced by the liver [63]. Zilebesiran is the first siRNA designed to reduce AGT mRNA in the hepatocytes, using a mechanism of delivery and RNAi like that of inclisiran and olpasiran (Figure 1). Liver-specific effects were demonstrated by both preclinical studies of a GalNAc-conjugated siRNA, which suggests near-complete knockdown of hepatic AGT mRNA without affecting renal AGT mRNA [64] and by clinical experience with GalNAc-conjugated antisense oligonucleotides targeting AGT [65]. With a published phase 1 study and promising phase 2 studies whose publication is expected in 2024, zilebesiran is also the first siRNA for the molecular therapy of essential hypertension, holding the promise of just two injections in a year, like inclisiran for anti-PCSK9 therapy. In a four-part phase 1 study [66], hypertensive patients were randomized (2:1 ratio) to either a single dose of zilebesiran (10, 25, 50, 100, 200, 400, or 800 mg) or placebo by the subcutaneous route and then followed for 24 weeks, in Part A. Part B evaluated the effect of the 800 mg dose of zilebesiran on BP under low- or high-salt diet regimens, and Part E the effect of that dose when co-administered with an angiotensin receptor blocker (irbesartan). Single doses of zilebesiran (≥200 mg) were associated with a clinically significant decrease in systolic blood pressure (SBP) (>10 mmHg) and diastolic blood pressure (DBP) (>5 mmHg) by week 8, as measured by 24-h ambulatory BP monitoring (ABPM). These changes were maintained throughout the diurnal cycle and sustained at 24 weeks. Results from Parts B and E were consistent with an attenuated effect on BP by a high-salt diet and, by contrast, with an augmented effect through coadministration with irbesartan, respectively. Serum AGT was reduced above 90% with 200 mg or more of zilebesiran and persisted low from week 3 to week 12 [66]. KARDIA-1 (NCT04936035) and KARDIA-2 (NCT05103332) are phase 2 studies aiming at investigating the potential of zilebesiran as antihypertensive therapy. Indeed, KARDIA-1 evaluated the efficacy and safety of monotherapy with zilebesiran in 378 adults affected by mild-to-moderate arterial hypertension, either untreated or during a stable treatment with one or more antihypertensive drugs. Patients have been randomly assigned to one of the following five treatment arms (150 mg or 300 mg every 6 months, 300 mg or 600 mg every 3 months, or placebo, by the subcutaneous route) during a double-blind period of 12 months and a double-blind extension period. After 6 months, patients taking a placebo were then randomly assigned to one of the four initial doses of zilebesiran. KARDIA-1 met its primary endpoint on 7 September 2023, proving a clinically significant lowering in mean 24 h-SBP after 3 months (*p* < 0.0001) with both zilebesiran doses versus placebo. KARDIA-1 also met key secondary endpoints. Indeed, all zilebesiran arms led to significant mean 24h-SBP lowering after 6 months and significant office SBP lowering after 3 and 6 months compared to placebo, thus highlighting the potent and durable inhibition of AGT exerted by this innovative drug. The KARDIA-2 trial will show the role of zilebesiran combined with another antihypertensive drug in mild-to-moderate hypertension, and the results are expected in early 2024. The clinically significant SBP lowering, coupled with the possibility of obtaining a “tonic” BP control with probably just two injections in a year, will help overcome the therapeutic adherence problem and resolve most cases of uncontrolled hypertension due to BP variability or lack of night-time control. The hepatocyte-targeted delivery preserves the extrahepatic AGT expression, limiting effects in the kidney and other tissues, such as the adipose tissue, that was believed to be a relevant source of AGT, especially in the obese [67]. The AGT gene is indeed expressed in the human kidney cortex and medulla and in visceral adipose tissue in a differential manner, influenced also by genetic variants [68]. In visceral perirenal adipose tissue, AGT expression is about five-fold higher than in kidney cortex and medulla, that express similar AGT mRNA levels [68]. The available data show that zilebesiran at higher dosages reduces AGT close to 100%, indicating that the contribution to circulating AGT levels from other cells, such as adipocytes, is very low, even if it can be locally relevant. Nevertheless, data presented at the “Hypertension 2023” American Heart Association Council on Hypertension September meeting in Boston, showed that in obese patients treated with zilebesiran results were similar to those previously found in the aforementioned trials [69]. KARDIA-1 also demonstrated the safety and tolerability of zilebesiran, thus supporting the continuation of its development; one patient in the zilebesiran arm died from cardiopulmonary arrest that was not defined related to the drug. Placebo-treated patients (6.7%) reported more serious adverse events than zilebesiran-treated patients (3.6%). The adverse events reported by 5% or more of any zilebesiran group were ISR, hyperkalemia, hypertension, upper airways infection, arthralgia, headache, and COVID-19. As a precaution in the eventual need to reverse the effect of zilebesiran (e.g., severe hypotension/hypovolemia/shock or pregnancy), a rapid and potent reversal of siRNA-mediated gene silencing has been developed using GalNAc-conjugated single-stranded oligonucleotide complementary to the siRNA guide strand. This oligonucleotide is rapidly taken up by the liver and blocks the siRNA activity of zilebesiran [70]. This is also an unprecedented progress in clinical CV pharmacology.

## 6. Conclusions

New molecular treatments for CV diseases are now a reality. The mechanisms of action, potential uses, and benefits of siRNAs reported in this review are summarized in Table 2. The siRNAs described in this focused review are just a good beginning. Adherence to therapy will no longer be a problem anymore. The safety of these modified and stabilized RNA molecules is very good to date. The cost of each new “revolutionary” treatment is certainly a concern at the beginning, but it must be balanced with the cost of CV events determined by uncontrolled LDL-C, Lp(a) and arterial BP levels. Furthermore, the mechanism of RNAi opens novel paths toward innovative therapies in many fields. Even though all the siRNAs that are currently used in CV clinical practice target the liver, there are several other siRNAs under investigation targeting different organs [71], such as brain for the therapy of glioblastoma [72], kidney for acute kidney injury [73], lung for COVID-19 (NCT05184127), and eye for glaucoma [74], age-related macular degeneration [75] and diabetic retinopathy (NCT01445899). siRNAs offer a promising treatment for a wide range of diseases other than the CV ones, including viral infections, cancer, metabolic, hematologic, neurodegenerative, and ocular diseases, using various conjugation systems and delivery mechanisms [76]. The progress in science and evidence-based medicine is what will bring us longer, healthy lives, also thanks to these novel, long-lasting twice-a-year therapies against the most common CV risk factors and diseases.

## Figures and Tables

**Table 1:** List of siRNAs reported in the text and main clinical trials conducted on them.

	Trial	Subjects and Sample Size (n)	Objectives	Results	Phase
	ORION-9	Subjects with HeFH and elevated LDL-C (482)	To evaluate the effect of inclisiran 300 mg compared to placebo in LDL-C lowering	A 39.7% (95% CI, −43.7 to −35.7) LDL-C lowering at day 510 with inclisiran	Phase III
	ORION-10	Subjects with established ASCVD (1561)	To evaluate the effect of inclisiran 284 mg compared to placebo in LDL-C lowering	A 52.3% (95% CI, 48.8 to 55.7) LDL-C lowering with inclisiran	Phase III
Inclisiran	ORION-11	Subjects with ASCVD equivalent risk (1617)	To assess efficacy and safety of inclisiran 284 mg compared to placebo in LDL-C lowering	A 49.2% (95% CI, 46.6 to 53.1) LDL-C lowering with inclisiran	Phase III
	HPS-4/TIMI 65/ORION-4	Subjects with established ASCVD (16124)	To evaluate if inclisiran 300 mg reduces the risk of MACE	Expected in July 2026	Phase III
	VICTORION-2P	Subjects with established ASCVD (16500)	To evaluate if inclisiran 300 mg reduces the risk of MACE	Expected in October 2027	Phase III
	NCT03626662	Subjects with elevated plasma Lp(a) (80)	To assess safety, tolerability, pharmacokinetics, and pharmacodynamic effects of olpasiran	A safe and persisting 71–97% reduction in Lp(a) concentration	Phase I
Olpasiran	OCEAN(a)-DOSE	Subjects with established ASCVD and a serum Lp(a) concentration of more than 150 nmol/L (~70 mg/dL) (281)	To evaluate the percent change in the Lp(a) concentration from baseline to week 36 with four different doses of olpasiran	Serum Lp(a) levels significantly reduced in a dose-dependent manner at 36 weeks	Phase II
	OCEAN(a)-Outcomes (NCT05581303)	Subjects with established ASCVD and elevated plasma Lp(a) (6000)	To evaluate if olpasiran reduces the risk of coronary heart disease death, myocardial infarction, or urgent coronary revascularization	Expected in December 2026	Phase III
Lepodisiran	NCT04914546	Subjects without CV disease and with a serum Lp(a) concentration of 75 nmol/L or greater (or ≥30 mg/dL)	To assess the safety, tolerability, pharmacokinetics, and pharmacodynamic effects of lepodisiran	A safe and persisting 41–94% reduction in Lp(a) concentration	Phase I
ARO-APOC3	NCT03783377	Healthy subjects and subjects with hypertriglyceridemia (112)	To assess the safety, tolerability, pharmacokinetics, and pharmacodynamic effects of ARO-APOC3	ARO-APOC3 associated with few adverse events and reduced serum levels of APOC3 and triglycerides	Phase I
	NCT01961921	Subjects with vATTR-polyneuropathy (27)	To assess safety, effects on serum TTR levels, and clinical parameters (mNIS + 7 and multiple disease-relevant measures) of patisiran treatment (0.3 mg/kg intravenously every 3 weeks)	No drug-related adverse events leading to treatment discontinuation, sustained reduction in mean transthyretin levels, a mean 6.95-point improvement in mNIS + 7 from baseline	Phase II
Patisiran	APOLLO	Subjects with vATTR-polyneuropathy (225); cardiac subpopulation with a left ventricular wall thickness ≥ 13 mm without history of hypertension or aortic valve disease (126)	To evaluate the effect of patisiran treatment (0.3 mg/kg intravenously every 3 weeks) on neurological symptoms compared to placebo	Least-squares mean (±SE) change from baseline of mNIS + 7 was −6.0 ± 1.7 versus 28.0 ± 2.6 (difference, −34.0 points; *p* < 0.001) at 18 months; effects also on gait speed and modified BMI; in the cardiac subpopulation, patisiran reduced mean left ventricular wall thickness (least-squares mean difference ± SEM: –0.9 ± 0.4 mm, *p* = 0.017), interventricular septal wall thickness, posterior wall thickness, and relative wall thickness; increased end-diastolic volume and reduced NT-proBNP. In a post-hoc analysis, patisiran treatment lowered combined all-cause hospitalization and mortality compared with placebo at month 18	Phase III
	APOLLO-B	Subjects with both wild-type and vATTR-cardiomyopathy with clinical evidence of HF and an elevated NT-proBNP between 300 ng/L and 8500 ng/L (360)	To evaluate the efficacy of patisiran treatment (0.3 mg/kg intravenously every 3 weeks) compared to placebo in patients with cardiomyopathy: change from baseline in the distance covered on 6MWT, change from baseline in the KCCQ-OS score, differences in death from any cause, CV events, hospitalizations for any cause, and urgent HF visits over 12 months	The decline in the 6MWT was lower in the patisiran group (95% CI, 0.69 to 28.69; *p* = 0.02); the KCCQ-OS score increased in the patisiran group and declined in the placebo group (least-squares mean difference, 3.7 points; 95% CI, 0.2 to 7.2; *p* = 0.04). Significant benefits were not observed for secondary endpoints (all-cause hospitalization, urgent HF visits, and death)	Phase III
Vutrisiran	HELIOS-A	Subjects with vATTR-polyneuropathy (164)	To evaluate the effect of vutrisiran 25 mg every 3 months on neurological symptoms compared to patisiran and placebo	Change from baseline in mNIS + 7 at 9 months (*p* = 3.54 × 10^−12^); significant improvements versus external placebo in Norfolk Quality of Life-Diabetic Neuropathy, 10-m walk test, mNIS + 7	Phase III
	HELIOS-B	Subjects with both wild-type and vATTR-cardiomyopathy (655)	To evaluate the efficacy and safety of vutrisiran 25 mg every 3 months compared to placebo in patients with ATTR amyloidosis and cardiomyopathy. Composite endpoint of all-cause mortality and recurrent CV events	Expected in early 2024	Phase III
	NCT03934307	Patients with arterial hypertension (107)	To assess safety, pharmacokinetics, and pharmacodynamic effects of single doses of zilebesiran (10, 25, 50, 100, 200, 400, or 800 mg), and the change from baseline in systolic and diastolic BP	Single doses of zilebesiran (≥200 mg) associated with decreases in systolic (>10 mmHg) and diastolic BP (>5 mmHg) by week 8 and sustained to week 24. Mild ISRs	Phase I
Zilebesiran	KARDIA-1	Subjects with a daytime mean systolic BP ≥135 mmHg and ≤160 mmHg (378)	To evaluate the efficacy of different doses of zilebesiran compared to placebo in patients with hypertension	Change in ambulatory systolic BP: –11.1 mmHg for zilebesiran 150 mg every 6 months vs. –14.5 mmHg for zilebesiran 300 mg every 6 months vs. –4.1 mmHg for zilebesiran 300 mg every 3 months vs. –14.2 mmHg for zilebesiran 600 mg every 6 months (*p* < 0.05 for each group)	Phase II
	KARDIA-2	Subjects with hypertension not adequately controlled by a standard of care antihypertensive medication (672)	To evaluate the efficacy of zilebesiran compared to placebo in patients with hypertension	Expected in early 2024	Phase II

HeFH: heterozygous familial hypercholesterolemia; LDL-C: low-density lipoprotein cholesterol; ASCVD: atherosclerotic cardiovascular disease; MACE: major adverse cardiovascular events; Lp(a): lipoprotein(a); APOC3: apolipoprotein C-III; HF: heart failure; vATTR: variant transthyretin amyloidosis; mNIS: modified Neuropathy Impairment Score; 6MWT: 6 min walking test; KCCQ: Kansas City Cardiomyopathy Questionnaire; BP: blood pressure; ISR: injection site reaction.

**Table 2:** List of siRNAs reported in the text, their mechanism of action, and potential uses, and benefits.

siRNA	Mechanism of Action	Potential Uses and Benefits
Inclisiran	PCSK9 mRNA degradation in the liver and inhibition of PCSK9 synthesis and secretion, leading to increased LDLR expression on the surface of hepatocytes and reduced circulating LDL-C levels	Significant, sustained and safe LDL-C lowering in patients at high and very high CV risk that do not reach their LDL-C goal with traditional lipid-lowering therapy (statins and ezetimibe) and/or are statin-intolerant. Potential reduction in the risk of MACE in patients at high and very high CV risk. Optimal adherence to therapy, with only two injections in a year
Olpasiran and Lepodisiran	LPA mRNA degradation in the liver and inhibition of Lp(a) assembly and secretion	Significant, sustained, and safe Lp(a) lowering in patients with high Lp(a) circulating levels. Potential reduction in the risk of ischemic CV disease, ASCVD, and calcific valvular aortic stenosis. Reduction in OxPL-apoB levels, a biomarker strongly associated with ASCVD. Optimal adherence to therapy, with only two or three injections in a year
ARO-APOC3	APOC3 mRNA degradation in the liver and inhibition of APOC3 synthesis and secretion, leading to increased triglycerides clearance and hepatocyte uptake of triglyceride-rich lipoproteins	Significant, sustained, and safe triglycerides lowering in patients with hypertriglyceridemia and chylomicronemia. Potential reduction in the risk of ASCVD and pancreatitis in patients with hypertriglyceridemia and chylomicronemia
Patisiran and Vutrisiran	TTR mRNA degradation in the liver and inhibition of TTR synthesis and secretion, thereby preventing its abnormal accumulation in amyloidosis	Significant, sustained, and safe TTR lowering in patients with ATTR amyloidosis. Improvement of neurological symptoms and quality of life in patients with ATTR-polyneuropathy. Improvement of cardiac parameters and symptoms in patients with ATTR-cardiomyopathy. Increase in survival in patients with ATTR amyloidosis. Potential reduction in all-cause mortality and CV events in patients with ATTR-cardiomyopathy. Optimal adherence to therapy, with only four injections in a year (only for vutrisiran)
Zilebesiran	AGT mRNA degradation in the liver and inhibition of AGT synthesis and secretion, thereby preventing the production of angiotensin peptides and inhibiting the renin-angiotensin-aldosterone system	Significant, sustained, and safe systolic and diastolic BP lowering in patients with uncontrolled hypertension. Reduction in the risk of CV events in patients with uncontrolled or resistant hypertension. Optimal adherence to therapy, with only two or four injections in a year

PCSK9: proprotein convertase subtilisin/kexin type 9; LDLR: low-density lipoprotein receptor; CV: cardiovascular; LDL-C: low-density lipoprotein cholesterol; MACE: major adverse cardiovascular events; LPA: apolipoprotein(a) gene; Lp(a): lipoprotein(a); ASCVD: atherosclerotic cardiovascular disease; APOC3: apolipoprotein C-III; ATTR: transthyretin amyloidosis; AGT: angiotensinogen; BP: blood pressure.

## Data Availability

Data are contained within the article.
